# Mediation of nutritional status between the number of teeth and depressive symptoms in multi-ethnic older adults from Western China

**DOI:** 10.3389/fpubh.2025.1506640

**Published:** 2025-04-29

**Authors:** Xin Tian, Xin Xia, Huixian Li, Yuexia Hu, Yuqing Xie, Xiuying Hu, Jirong Yue, Birong Dong, Bei Wu, Yanyan Wang

**Affiliations:** ^1^Healthcare Innovation Research Laboratory, West China School of Nursing & National Clinical Research Center for Geriatrics, Science and Technology Department, West China Hospital, Sichuan University, Chengdu, China; ^2^Department of Geriatrics and National Clinical Research Center for Geriatrics, West China Hospital, Sichuan University, Chengdu, China; ^3^Innovation Center of Nursing Research and Nursing Key Laboratory of Sichuan Province, West China Hospital, Sichuan University/West China School of Nursing, Sichuan University, Chengdu, China; ^4^Rory Meyers College of Nursing and NYU Aging Incubator, New York University, New York, NY, United States

**Keywords:** the number of teeth, depressive symptoms, nutritional status, mediation analysis, older adults

## Abstract

**Background:**

Depression is a prevalent issue among older adults, affecting the quality of life and overall health of individuals. This study aimed to investigate the role of nutritional status in mediating the number of teeth and depressive symptoms.

**Method:**

A prospective multi-ethnic baseline data of 6,632 adults aged 50 years and older was derived from the 2018 West China Health and Aging Trend study. Depressive symptoms were assessed using the 15-item Geriatric Depression Scale, and nutritional status was evaluated with the Mini Nutritional Assessment-Short Form. A multiple linear regression was performed to assess the associations among the number of teeth, nutritional status, and depressive symptoms. Mediation models and pathway analysis were employed to investigate the mediating role of nutritional status.

**Results:**

The sample comprised 18 ethnic groups from western China. The percentage of depressive symptoms among participants was 17.3%. Multiple linear regression indicated a significant correlation between the number of teeth and depressive symptoms. The association remained statistically significant (β = −0.089; 95% CI −0.158, −0.020) after adjusting for MNA-SF scores. Mediation analysis confirmed nutritional status partially mediated the relationship between the number of teeth and depressive symptoms (indirect effect estimate = −0.059; 95% CI −0.076, −0.044, direct effect estimate = −0.089; 95% CI −0.158, −0.020). Furthermore, structural equation model for pathway analysis verified the correlation between the number of teeth, nutritional status, and depressive symptoms (*p* < 0.05).

**Conclusion:**

Nutritional status partially mediated the association between the number of teeth and depressive symptoms, revealing significant direct and indirect effects. Early identification of nutritional deficits and the maintenance of oral health are essential for preventing depression in older adults.

## 1 Introduction

With the extension of human life expectancy, aging has emerged as a significant public health challenge confronting the world (1). As individuals age, they inevitably suffer from a series of health problems, including cardiovascular diseases, metabolic disorders, neurodegenerative diseases, as well as mental disorders (2). A meta-analysis reported that depressive symptoms impact around 20% of older adults across China (3). However, cultural factors, such as the Confucian belief in accepting fate and the under-recognition in this population may lead to an underestimation of its true prevalence (4). Depression leads to higher risk of cardiovascular diseases, suicide (5), and exacerbation of comorbidities (6). It is reported that the total direct costs for older adults with depression are 73% higher compared to those without depression (7). Identifying the risk factors of depressive symptoms is crucial to developing tailored interventions. Previous studies indicated that oral health and nutritional status are associated with depression among older adults in several countries, such as the U.S., and the UK (8–10). However, less research focuses on the relationship between the number of teeth and depressive symptoms mediated by nutritional status among older adults.

Oral health is a critical determinant of overall health among older adults (11). Previous studies have indicated that poor oral health is associated with a higher risk of depression (12, 13) in the older population. The number of teeth serves as an objective indicator reflecting oral health. An increasing number of studies have indicated a correlation between the number of teeth and depression, malnutrition, and quality of life in aging individuals (14–19). Tooth loss is a prevalent issue among older adults (20). It is reported that nearly 50% of individuals aged 65 years and older in China experience unrepaired missing teeth (21). Tooth loss impacts oral function, orofacial appearance, speech ability, and self-esteem of older adults, subsequently leading to social isolation and depression (22).

Malnutrition is also prevalent among older adults, affecting 41.2% of community-dwelling individuals aged 60 years and older in China (23). Malnutrition in this population leads to adverse health outcomes, including depression, frailty, and sarcopenia (24, 25). Furthermore, malnutrition increases the risk of depression, which may be attributed to various nutrients (26). Nutrients such as omega-3 fatty acids, B vitamins (especially B12 and folate), vitamin C, magnesium, and zinc are essential for the maintenance of brain health. Deficiencies in these nutrients could affect the synthesis and function of neurotransmitters, which are associated with depressive symptoms (27). The number of teeth is closely associated with the nutritional status of older adults. A decreased number of teeth could impact their chewing ability, potentially leading to alterations to dietary preferences and affecting their overall nutritional intake (28), ultimately resulting in poor nutrition (29). The population of ethnic minorities in China is approximately 125 million, with the highest proportion located in the Western region, accounting for 70.22% (30). Distinct ethnic groups possess diverse genetic backgrounds, lifestyles, and dietary habits, which significantly influence their oral health, nutritional status, and vulnerability to depressive symptoms (31, 32). The mediating role of nutritional status between the number of teeth and depressive symptoms has not been elucidated among the multi-ethnic older population in Western China. Therefore, this study aimed to investigate whether nutritional status mediates the relationship between the number of teeth and depressive symptoms among multi-ethnic older adults in western China, as well as to determine the extent of this mediation contribution. The findings from this research could provide a scientific basis for tailored health interventions.

## 2 Materials and methods

### 2.1 Participants and procedures

**Figure 1:**
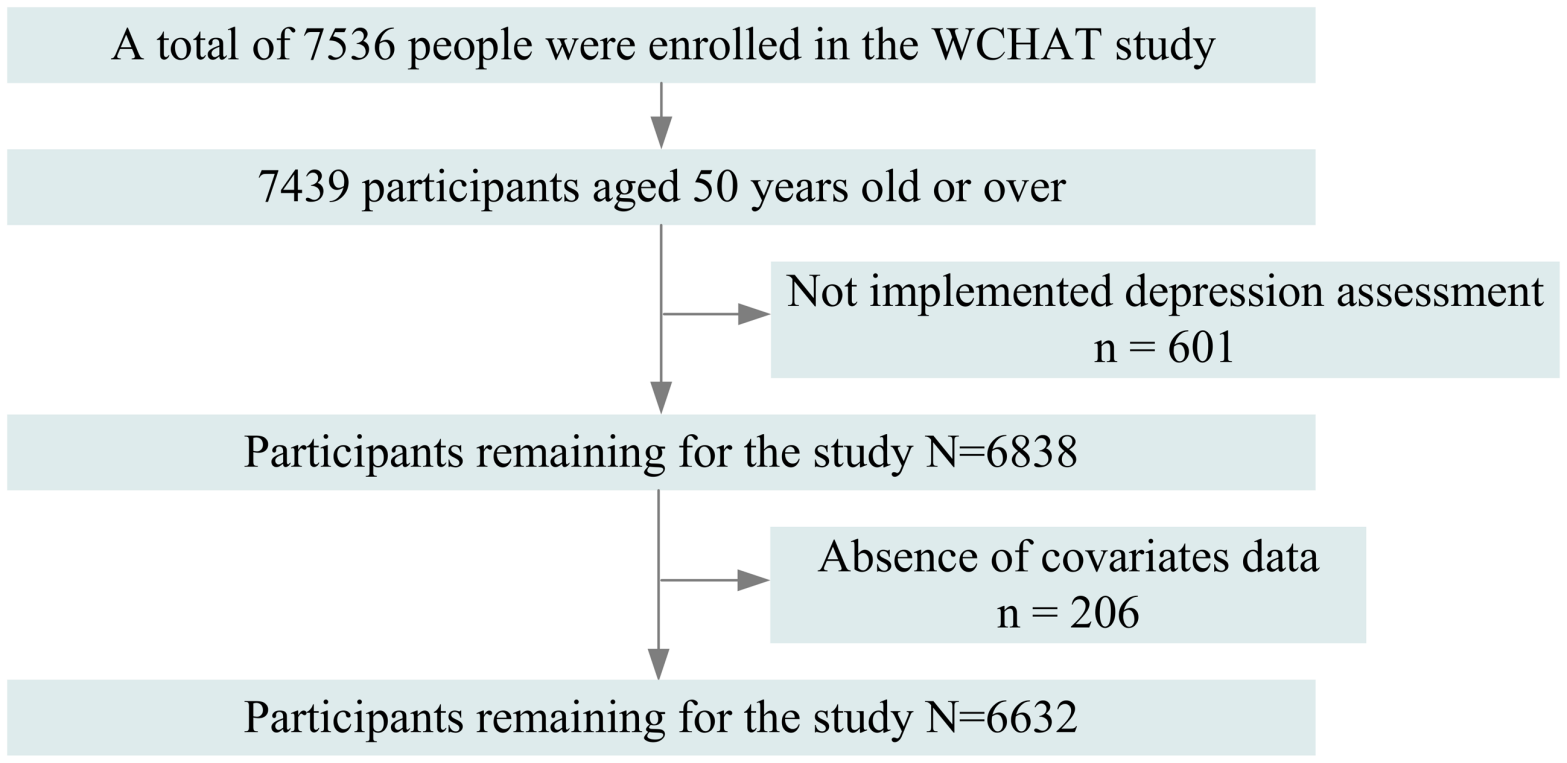
Flow chart of study participants.

The baseline data for the cross-sectional analysis were drawn from the 2018 wave of the West China Health and Aging Trend (WCHAT), a prospective multi-ethnic cohort study. A multi-stage random cluster sampling scheme was employed (33). The data were collected from 7,536 individuals representing 18 ethnic groups across four ethnically diverse regions of China: Xinjiang, Sichuan, Guizhou, and Yunnan by 7 medical institutions including (1) The West China Hospital of Sichuan University; (2) The Fifth People’s Hospital of Chengdu; (3) The Eighth People’s Hospital of Chengdu; (4) The Fifth Affiliated Hospital of Xinjiang Medical University; (5) The Yan’an Affiliated Hospital of Kunming Medical University; (6) The First Affiliated Hospital of Kunming Medical University; and (7) The Guizhou Province People’s Hospital. All researchers involved in data collection underwent one-on-one training and assessment before the investigation. The questionnaire adapted to reflect the cultural, linguistic, and lifestyle characteristics of western China. Details of the cohort had been published elsewhere (33). The inclusion criteria established in this study: (1) those who were 50 years and older; (2) those who implemented the depression assessment; (3) those who were no absence of covariates data. Participants who were under 50 years old (*n* = 97), did not implement depression assessment (*n* = 601), and lacked covariates data (*n* = 206) were excluded from the study. Ultimately, this study included 6,632 participants in analysis (Figure 1).

The study received approval from the Ethical Review Committee of Sichuan university (reference: 2017-445) and registered in the Chinese Clinical Trial Registry (number: 180018895). The research adhered to the principles outlined in the Declaration of Helsinki.

### 2.2 Measurements

#### 2.2.1 The number of teeth

The number of teeth was examined and counted by professional dentists. All dentists underwent standardized training and successfully passed a certification assessment before participating in the evaluation process. At each investigation site, two dentists conducted dental examinations simultaneously to ensure consistency. Whether individuals have denture usage was also inquired and checked.

#### 2.2.2 Depressive symptoms

The depressive symptoms were evaluated employing the 15-item Geriatric Depression Scale (GDS-15), which consisted of 15 binary questions assessing feelings over the past week. The total scores ranged from 0 to 15, with a Cronbach’s alpha of 0.894 (34). A GDS-15 score of 5 or higher is indicative of depressive symptoms (35). This scale is widely utilized for detecting depression in older Chinese adults (36).

#### 2.2.3 Nutritional status

The nutritional status of individuals was measured by the Mini Nutrition Assessment-Short Form (MNA-SF) scale, which comprised six items with an overall score ranging from 0 to 14. The Cronbach’s alpha of this 6-item MNA-SF scale was 0.87 (37), indicating high internal consistency. The 6 items include: (1) Has food intake declined over the past 3 months due to loss of appetite, digestive problems, chewing or swallowing difficulties? (2) Weight loss during the last 3 months; (3) Mobility; (4) Has suffered psychological stress or acute disease in the past 3 months? (5) Neuropsychological problems; (6) Body Mass index (BMl) (weight in kg) / (height in m^2^). If BMI is not available, replace it with calf circumstance (CC) in cm (38). It has been validated as an effective tool for screening older adults for the risk of malnutrition in various settings. The MNA-SF classified nutritional status as follows: 0–7 indicated malnutrition, 8–11 suggested a risk of malnutrition, and 12–14 denoted good nutritional status (39).

#### 2.2.4 Covariates

Demographic, health-related and behavioral characteristics were adjusted in all models. Demographic characteristics include age, educational level, gender, marital status, occupation, longevity families (at least one family member aged 90 years or older across four generations), and ethnic group. Health-related characteristics encompass denture usage. Behavioral characteristics contain the type of drinking water, history of smoking, alcohol use, and tea drinking. These variables were comparable between groups.

### 2.3 Statistical analysis

Variables are described using the mean, standard deviation (SD), and percentage based on variable type. Differences between depressive symptoms categories and the variables under study were analyzed by analysis of variance (for continuous variables) and chi-square tests (for categorical variables). Utilizing multiple linear regression to investigate the correlation between the number of teeth, nutritional status, and depressive symptoms. Three multiple regression models were used to estimate 95% confidence intervals (CI) for each model. The number of teeth was used as the independent variable in all three models. Model 1 adjusted for age, educational level, gender, marital status, occupation, longevity families, religious belief, and ethnic group, denture usage status, and behavioral characteristics. Model 2 incorporated adjustments from model 1 and nutritional status scores. Two models used depressive symptoms scores as the dependent variable. Model 3 used nutritional status scores as the outcome variable with the same adjustment variables as model 1. The potential mediating effect of nutrition status on the relationship between the number of teeth and depressive symptoms was investigated using the total score of MNA-SF as a multi-categorical mediating model. Performing path analysis within the structural equation modeling (SEM) framework. All analyses were performed using R 4.3.0 with statistical testing set at two-sided significance value *p* < 0.05.

## 3 Results

### 3.1 Baseline characteristics

Overall, this study included a total of 6,632 participants, consisting of 4,147 women and 2,485 men who were aged 50 years old or older. The age of participants ranged from 50 to 95 years. Table 1 displays the demographic characteristics of the participants categorized by depressive symptoms. A total of 1,148 (17.3%) participants exhibited depressive symptoms, with a higher prevalence among women. Participants who were single, occasionally toothbrush usage, no usage of toothpaste (*p* < 0.05), lower educational level, and had poorer self-assessed oral health (*p* < 0.001) exhibited a higher prevalence of depressive symptoms. Additionally, participants with no history of smoking and drinking tea showed a higher prevalence of depressive symptoms (*p* < 0.001). The mean number of teeth was 21.2 (standard deviation [SD] 9.5), while the average MNA-SF score was 12.6 (SD 1.6). Supplementary Figure S1 illustrates the distribution of the number of teeth in different ethnic groups, whereas Supplementary Figure S2 shows the proportion of the number of teeth and depressive symptoms in different ethnic groups. Furthermore, it was observed that participants exhibiting depressive symptoms had a lower number of teeth (21.3 [9.4] vs. 20.6 [9.9]; *p* < 0.05) and lower MNA-SF scores (12.7 [1.5] vs. 12.1 [1.8]; *p* < 0.001) compared to normal group. This indicates that depressed individuals may have fewer residual teeth and poorer nutritional status.

**Table 1:** Sample characteristics grouped by depressive symptoms.

Characters	Total *N* = 6,632	Normal *N* = 5,484	Depressive symptoms *N* = 1,148	*p* value
Main variables				
Number of teeth, mean (SD)	21.2 (9.5)	21.3 (9.4)	20.6 (9.9)	0.020
Number of teeth, *n* (%)				0.027
0–8	993 (15.0)	799 (80.5)	194 (19.5)	
9–16	724 (10.9)	580 (80.1)	144 (19.9)	
17–24	1,511 (22.8)	1,263 (83.6)	248 (16.4)	
25–32	3,404 (51.3)	2,842 (83.5)	562 (16.5)	
MNA-SF score, mean (SD)	12.6 (1.6)	12.7 (1.5)	12.1 (1. 8)	<0.001
MNA-SF assessment status, *n* (%)				<0.001
Normal	5,184 (78.2)	4,392 (84.7)	792 (15.3)	
Nutrition risk	1,392 (21.0)	1,055 (75.8)	337 (24.2)	
Malnutrition	56 (0.8)	37 (66.1)	19 (33.9)	
Covariates				
Demographic characteristics				
Age, mean (SD)	62.4 (8.3)	62.3 (8.2)	62.7 (8.5)	0.135
Age group, *n* (%)				0.240
50–59	2,632 (39.7)	2,191 (83.2)	441 (16.8)	
60–69	2,642 (39.8)	2,192 (83.0)	450 (17.0)	
70–79	1,174 (17.7)	947 (80.7)	227 (19.3)	
80+	184 (2.8)	154 (83.7)	30 (16.3)	
Gender, *n* (%)				<0.001
Men	2,485 (37.5)	2,135 (85.9)	350 (14.1)	
Women	4,147 (62.5)	3,349 (80.8)	798 (19.2)	
Ethnic group, *n* (%)				<0.001
Han	2,393 (36.1)	2,063 (86.2)	330 (13.8)	
Zang	1,276 (19.2)	1,080 (84.6)	196 (15.4)	
Qiang	1,272 (19.2)	963 (75.7)	309 (24.3)	
Yi	606 (9.1)	478 (78.9)	128 (21.1)	
Uyghur	563 (8.5)	488 (86.7)	75 (13.3)	
Other	522 (7.9)	412 (78.9)	110 (21.1)	
Educational level, *n* (%)				<0.001
No formal education	1,842 (27.8)	1,422 (77.2)	420 (22.8)	
Elementary school	2,237 (33.7)	1,827 (81.7)	410 (18.3)	
Middle school	1,437 (21.7)	1,241 (86.4)	196 (13.6)	
High school and above	1,116 (16.8)	994 (89.1)	122 (10.9)	
Occupation, *n* (%)				<0.001
Farmer	4,320 (65.1)	3,439 (79.6)	881 (20.4)	
Worker	530 (8.0)	454 (85.7)	76 (14.3)	
White-collar	696 (10.5)	618 (88.8)	78 (11.2)	
Others	1,086 (16.4)	973 (89.6)	113 (10.4)	
Marital status, *n* (%)				0.041
Without spouse	1,098 (16.6)	884 (80.5)	214 (19.5)	
With Spouse	5,534 (83.4)	4,600 (83.1)	934 (16.9)	
Religious belief, *n* (%)				0.465
No	4,964 (74.8)	4,115 (82.9)	849 (17.1)	
Yes	1,668 (25.2)	1,369 (82.1)	299 (17.9)	
Longevity families, *n* (%)				<0.001
No	5,543 (83.6)	4,543 (82.0)	1,000 (18.0)	
Yes	1,089 (16.4)	941 (86.4)	148 (13.6)	
Health-related characteristics				
Self-assessed oral health, *n* (%)				< 0.001
Non-poor	4,942 (74.5)	4,161 (84.2)	781 (15.8)	
Poor	1,690 (25.5)	1,323 (78.3)	367 (21.7)	
Denture usage, *n* (%)				0.254
No	4,110 (62.0)	3,381 (82.3)	729 (17.7)	
Yes	2,522 (38.0)	2,103 (83.4)	419 (16.6)	
Toothbrush usage, *n* (%)				0.002
Never	493 (7.4)	398 (80.7)	95 (19.3)	
Occasionally	497 (7.5)	385 (77.5)	112 (22.5)	
Everyday	5,642 (85.1)	4,701 (83.3)	941 (16.7)	
Toothpicks usage, *n* (%)				0.231
Never	3,733 (56.3)	3,089 (82.7)	644 (17.3)	
Occasionally	1,173 (17.7)	952 (81.2)	221 (18.8)	
Everyday	1,726 (26.0)	1,443 (83.6)	283 (16.4)	
Toothpaste usage, *n* (%)				0.007
No	703 (10.6)	555 (78.9)	148 (21.1)	
Yes	5,929 (89.4)	4,929 (83.1)	1,000 (16.9)	
History of dental treatment, *n* (%)				0.108
Hadn’t	3,199 (48.2)	2,620 (81.9)	579 (18.1)	
Had	3,433 (51.8)	2,864 (83.4)	569 (16.6)	
Latest time of dental care, *n* (%)				0.356
Never	3,199 (48.2)	2,620 (81.9)	579 (18.1)	
More than 12 months	2,588 (39.0)	2,155 (83.3)	433 (16.7)	
Between 6 and 12 months	340 (5.1)	282 (82.9)	58 (17.1)	
Less than 6 months	505 (7.6)	427 (84.6)	78 (15.4)	
Reasons for latest dental care, *n* (%)				0.340
Not seeing a dentist	5,787 (87.3)	4,775 (82.5)	1,012 (17.5)	
Therapy	775 (11.7)	647 (83.5)	128 (16.5)	
Consultation or prevention	70 (1.1)	62 (88.6)	8 (11.4)	
Teeth cleaning, *n* (%)				0.185
No	6,202 (93.5)	5,139 (82.9)	1,063 (17.1)	
Yes	430 (6.5)	345 (80.2)	85 (19.8)	
Disease comorbidity, *n* (%)				0.962
No chronic disease	3,725 (56.2)	3,084 (82.8)	641 (17.2)	
One chronic disease	1,395 (210)	1,153 (82.7)	242 (17.3)	
Multiple chronic diseases	1,512 (22.8)	1,247 (82.5)	265 (17.5)	
Behavioral characteristics				
Type of drinking water, *n* (%)				<0.001
Tap water	5,316 (80.2)	4,457 (83.8)	859 (16.2)	
Well water	410 (6.2)	336 (82.0)	74 (18.0)	
Spring water	716 (10.8)	542 (75.7)	174 (24.3)	
Others	190 (2.9)	149 (78.4)	41 (21.6)	
History of smoking, *n* (%)				< 0.001
No	5,352 (80.7)	4,374 (81.7)	978 (18.3)	
Yes	1,280 (19.3)	1,110 (86.7)	170 (13.3)	
History of alcohol use, *n* (%)				0.193
No	4,910 (74.0)	4,042 (82.3)	868 (17.7)	
Yes	1,722 (26.0)	1,442 (83.7)	280 (16.3)	
History of drinking tea, *n* (%)				< 0.001
No	3,686 (55.6)	2,994 (81.2)	692 (18.8)	
Yes	2,946 (44.4)	2,490 (84.5)	456 (15.5)	

SD, standard deviation; MNA-SF, Mini Nutrition Assessment-Short Form.

Chronic diseases include hypertension, coronary heart disease, chronic obstructive pulmonary disease, gastrointestinal diseases, liver disease, kidney disease, diabetes, mental diseases, osteoarthritis, cancer, cataract, deafness, and so on. Other ethnic group including the Zhuang, Man, Hui, Mongolian, Tujia, Bai, Khalkhas, Dong, Miao, and Lisu ethnic groups.

### 3.2 Associations between the number of teeth and nutritional status, depressive symptoms

Table 2 presents the outcomes of the multiple regression analyses for three different models. The multiple linear regression analysis revealed a negative correlation between the number of remaining teeth and depressive symptoms (model 1 β = −0.148; 95% CI −0.218, −0.078; *p* < 0.001). The association remained significant (model 2 β = −0.089; 95% CI −0.158 to −0.020; *p* < 0.05) even after adjusting for MNA-SF scores, although the coefficient of association decreased. Furthermore, the correlation between the number of teeth and nutritional status was statistically significant (model 3 β = 0.197; 95% CI 0.153, 0.241; *p* < 0.001).

**Table 2:** Multiple linear regression analysis of depressive symptoms, the number of teeth, and nutritional status.

Outcome variable		Model 1: Depressive symptoms			Model 2: Depressive symptoms			Model 3: MNA-SF score		
		β	*p*	95%CI	β	*p*	95%CI	β	*p*	95%CI
MNA-SF score		–	–	–	−0.301	< 0.001	−0.338 to −0.263	–	–	–
Number of teeth		−0.148	< 0.001	−0.218 to −0.078	−0.089	0.012	−0.158 to −0.020	0.197	< 0.001	0.153 to 0.241
Age group	50–59	Ref			Ref			Ref		
	60–69	−0.176	0.012	−0.314 to −0.038	−0.163	0.018	−0.299 to −0.028	0.043	0.332	−0.044 to 0.130
	70–79	−0.046	0.634	−0.234 to 0.142	−0.126	0.180	−0.311 to 0.058	−0.268	< 0.001	−0.387 to −0.150
	80+	−0.285	0.147	−0.671 to 0.100	−0.424	0.028	−0.803 to −0.045	−0.461	< 0.001	−0.704 to −0.219
Gender	Men	Ref			Ref			Ref		
	Women	0.194	0.017	0.034 to 0.354	0.159	0.048	0.002 to 0.316	−0.118	0.022	−0.218 to −0.017
Ethnic group	Han	Ref			Ref			Ref		
	Zang	0.114	0.208	−0.064 to 0.292	0.065	0.464	−0.109 to 0.240	−0.163	0.004	−0.275 to −0.051
	Qiang	0.693	< 0.001	0.521 to 0.865	0.681	< 0.001	0.512 to 0.850	−0.039	0.478	−0.147 to 0.069
	Yi	0.430	< 0.001	0.207 to 0.653	0.237	0.035	0.017 to 0.458	−0.641	< 0.001	−0.781 to −0.500
	Uyghur	0.350	0.005	0.108 to 0.592	0.240	0.048	0.002 to 0.478	−0.366	< 0.001	−0.518 to −0.213
	Others	0.883	< 0.001	0.652 to 1.114	0.715	< 0.001	0.487 to 0.943	−0.560	< 0.001	−0.705 to −0.415
Educational level	No formal education	Ref			Ref			Ref		
	Elementary school	−0.288	< 0.001	−0.441 to −0.135	−0.161	0.037	−0.312 to −0.010	0.421	< 0.001	0.325 to 0.517
	Middle school	−0.484	< 0.001	−0.668 to −0.301	−0.346	< 0.001	−0.527 to −0.165	0.459	< 0.001	0.344 to 0.575
	High school and above	−0.486	< 0.001	−0.724 to −0.249	−0.393	0.001	−0.626 to −0.159	0.312	< 0.001	0.162 to 0.462
Occupation	Farmer	Ref			Ref			Ref		
	Worker	−0.249	0.032	−0.477 to −0.021	−0.238	0.037	−0.462 to −0.015	0.036	0.620	−0.107 to 0.180
	White-collar	−0.438	0.001	−0.689 to −0.187	−0.366	0.004	−0.612 to −0.120	0.240	0.003	0.082 to 0.397
	Others	−0.433	< 0.001	−0.608 to −0.258	−0.390	< 0.001	−0.562 to −0.218	0.143	0.011	0.033 to 0.253
Marital status	Without spouse	Ref			Ref			Ref		
	Have spouse	−0.044	0.598	−0.209 to 0.120	−0.016	0.848	−0.178 to 0.146	0.095	0.073	−0.009 to 0.198
Longevity of family	No	Ref			Ref			Ref		
	Yes	−0.287	< 0.001	−0.445 to −0.129	−0.273	0.001	−0.428 to −0.118	0.048	0.347	−0.052 to 0.147
Denture usage	No	Ref			Ref			Ref		
	Yes	−0.183	0.013	−0.328 to −0.038	−0.136	0.062	−0.278 to 0.007	0.156	0.001	0.065 to 0.248
Type of drinking water	Tap water	Ref			Ref			Ref		
	Well water	0.264	0.035	0.018 to 0.509	0.273	0.026	0.032 to 0.514	0.031	0.695	−0.124 to 0.185
	Spring water	0.460	< 0.001	0.267 to 0.654	0.426	< 0.001	0.236 to 0.616	−0.114	0.067	−0.235 to 0.008
	Others	−0.121	0.501	−0.472 to 0.231	−0.079	0.653	−0.425 to 0.266	0.138	0.223	−0.084 to 0.359
History of smoking	No	Ref			Ref			Ref		
	Yes	−0.241	0.011	−0.427 to −0.055	−0.31	0.001	−0.493 to −0.128	−0.231	< 0.001	−0.348 to −0.114
History of alcohol use	No	Ref			Ref			Ref		
	Yes	0.013	0.866	−0.140 to 0.167	0.029	0.704	−0.121 to 0.180	0.053	0.282	−0.044 to 0.150
History of drinking tea	No	Ref			Ref			Ref		
	Yes	−0.126	0.054	−0.255 to 0.002	−0.069	0.280	−0.196 to 0.057	0.189	< 0.001	0.108 to 0.270
Intercept		3.469	< 0.001	3.079 to 3.860	6.994	< 0.001	6.410 to 7.578	11.723	< 0.001	11.478 to 11.969
Observations		6,632			6,632			6,632		
*R* ^2^		0.064			0.100			0.095		
Adjusted R^2^		0.061			0.094			0.092		
Residual standard error		2.394			2.350			1.506		
F statistic ([df; *p* value])		18.22 ([25, 6,606]; *p*<0.001)			27.60([26, 6,605]; *p*<0.001)			27.89 ([25, 6,606]; *p*<0.001)		

Cl, confidence interval; MNA-SF, Mini Nutrition Assessment-Short Form; Ref, reference group; df, degree of freedom.

Model 1: multiple linear regression analysis between the number of teeth and depressive symptoms.

Model 2: multiple linear regression analysis between the number of teeth and depressive symptoms adjusted by MNA-SF score.

Model 3: multiple linear regression analysis between number of teeth and MNA-SF score.

### 3.3 Mediation effect of nutritional status between the number of teeth and depressive symptoms

**Figure 2:**
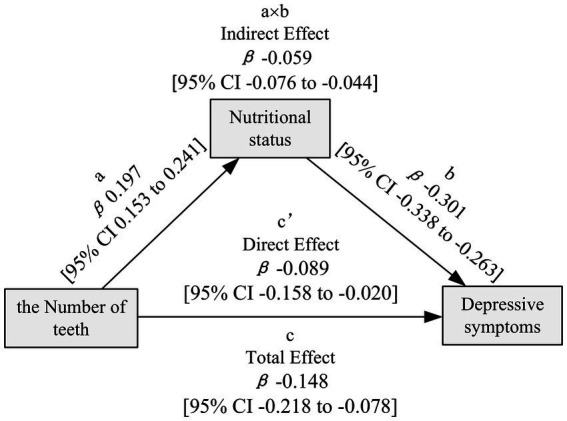
The mediating effect of nutritional status in the mediation model chart.

The study utilized mediating analysis to investigate the mediating effect of nutritional status. Figure 2 demonstrates the correlation between the number of teeth and depressive status mediated by nutritional status. The analysis of the mediation model revealed that lower number of teeth is associated with poorer nutritional status (β = 0.197; 95% CI 0.153, 0.241), and nutritional status is negatively correlated with depressive symptoms (β = −0.301; 95% CI −0.338, −0.263). The number of teeth was found to have statistically significant mediating effects (β = −0.059; 95% CI −0.076, −0.044), direct effects (β = −0.089; 95% CI −0.158, −0.020), and total effects (β = −0.148; 95% CI −0.218, −0.078) on depressive symptoms, which are mediated by nutritional status. This suggests that nutritional status partially mediates the link between the number of teeth and depressive symptoms. The mediating proportion of nutritional status was 40.12% (95% CI 25.23%, 77.75%) (Supplementary Table S1). Statistical details of the mediation analyses were adjusted for covariates in Supplementary Table S1.

### 3.4 Pathway analysis from the number of teeth and nutritional status to depressive symptoms

**Figure 3:**
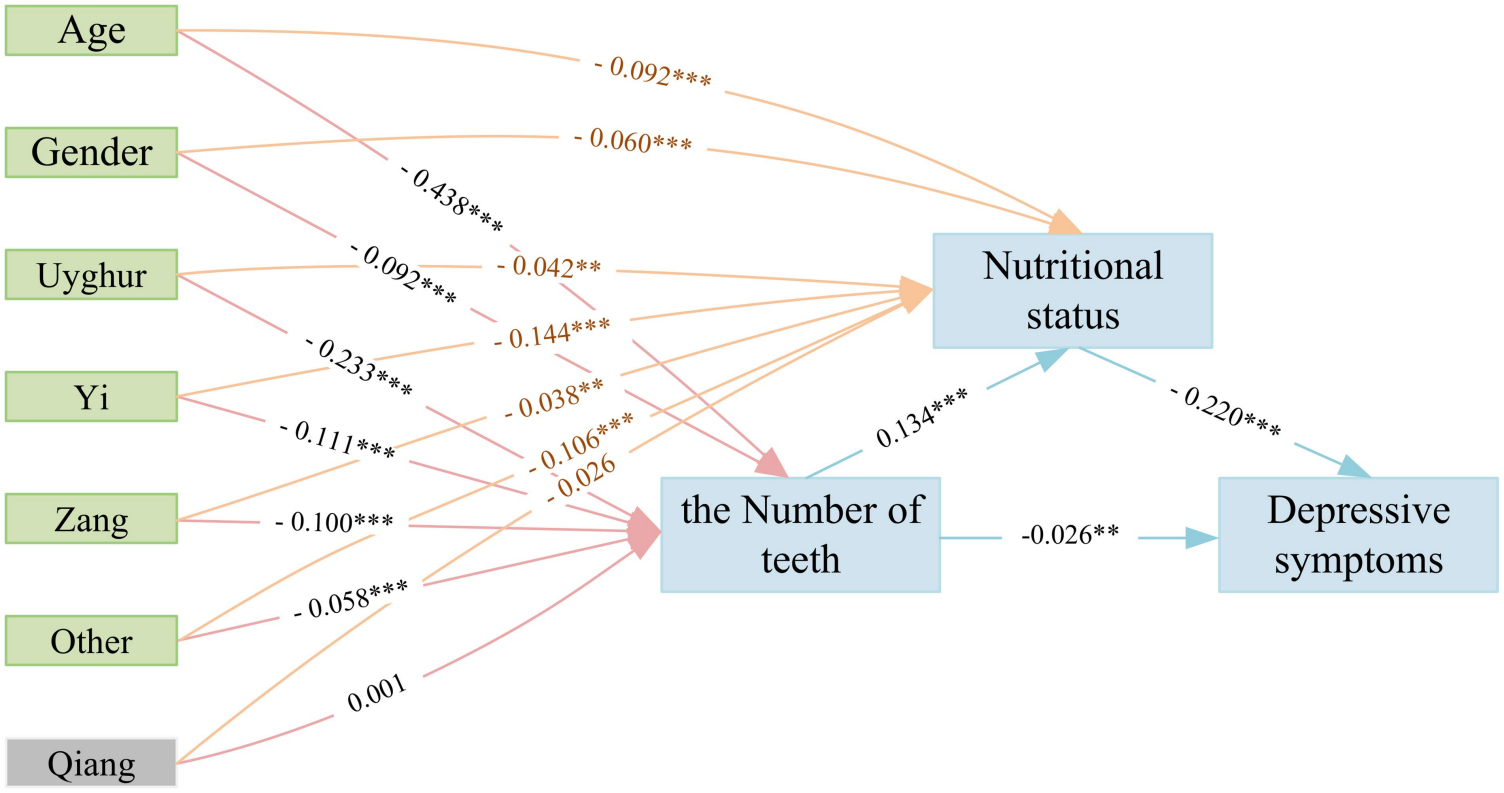
A pathway analysis of the nutritional status’s mediation effects using the structural equation model (SEM) framework. Other ethnic group including the Zhuang, Man, Hui, Mongolian, Tujia, Bai, Khalkhas, Dong, Miao, and Lisu ethnic groups (**p < 0.05; ***p < 0.001).

The structural equation modeling (SEM) framework was performed for pathway analysis (Figure 3; Supplementary Table S2). The pathway analysis using SEM revealed a negative correlation between the number of teeth and depressive symptoms (SEM coefficient: −0.026) and positively associated with nutritional status (SEM coefficient: 0.134), nutritional status was negatively related to depressive symptoms (SEM coefficient: −0.220). Furthermore, age and gender exhibited negative estimate coefficients in comparison to the number of teeth and nutritional status. Among the various ethnic groups, all except the Qiang displayed negative estimated coefficients when comparing the number of teeth and nutritional status. The Uygur group showing the most significant correlation with the number of teeth (SEM standardized coefficient: −0.233). Moreover, the Yi group demonstrated a strong coefficient between ethnicity and nutritional status (SEM normalization coefficient: −0.144). With regards to covariates, all *p*-values within the SEM pathway were significant except for the Qiang group. These findings further substantiated the relationship between the number of teeth, nutritional status, and depressive symptoms.

## 4 Discussion

This study indicated that the number of teeth is associated with nutritional status and depressive symptoms among multi-ethnic adults aged 50 and older in western China. Furthermore, nutritional status mediates in the relationship between the number of teeth and depressive symptoms, and the mediation effect was 40.12%.

We found an association between the number of teeth and depressive symptoms among older adults, which is consistent with previous research findings. A longitudinal study revealed that older adults with 19 or fewer teeth had a 3.9% higher prevalence of depressive symptoms during the follow-up period compared to those with 20 or more teeth (22). Mohammad et al. found that individuals with depression were 48% higher likelihood of being edentulous compared to those without depression (40). A population-based cohort study of 1,668 subjects reported that fewer number of teeth was linked to an increased risk of developing depressive symptoms in middle-aged and older individuals living in the community (41). Osteocalcin is a vitamin K-dependent protein that may play a role in the pathogenesis of tooth loss and depressive symptoms (42–44). However, the underlying mechanism linking the number of teeth to depressive symptoms remain unclear and require further investigation.

This study found that nutritional status partially mediated the relationship between the number of teeth and depressive symptoms through a mediation model. Previous studies have indicated that older adult individuals with fewer teeth are related to poorer nutritional status (45), which is consistent with this study. A recent systematic review revealed that older adults with edentulous or lacked functional dentition was associated with higher prevalence of malnutritional (46). The number of teeth affects individuals’ chewing ability, leading to restricted dietary choices and nutrients intake. Sheiham et al. (47) found a decrease in the number of teeth is linked to reduced intake of nutrients such as protein, vitamin C, folic acid, and serum β-carotene. Additionally, older adults with diminished chewing ability tend to consume more sugars, sweets, fats, and carbohydrates, instead of fresh vegetables and fruits, which are rich in vitamins (20, 45), thereby increase the risk of malnutrition. The dietary preferences of various ethnic groups may contribute to tooth loss, subsequently resulting in malnutrition (48). Previous systematic body of evidence indicates that nutritional status is associated with depressive symptoms (49, 50). A study on older rural adults indicated that those who were malnourished had around three times higher risk of depression (51) compared to well-nourished individuals. Data from older adults residing in community with low education levels revealed a correlation between poor nutritional status and depression (52). Cytokines, such as Growth differentiation factor-15 (GDF-15), may play a role in the pathogenesis of malnutrition and depression (53, 54). Related study found that GDF-15 levels independently predict nutritional status (53), with higher GDF-15 levels indicating poorer nutritional status. And depression in later life is associated with elevated GDF-15 levels (54), monitoring the GDF-15 levels of older adults are crucial for future research into the relationship between nutrition and depression. Related studies also clarified that the number of teeth, overall healthy dietary patterns and depression were related to brain volume (55). The potential mechanism of changes in brain volume may further support the association between the number of teeth, nutritional status, and depressive symptoms.

Previous research has shown that age and gender are correlated with both the number of teeth and nutritional status (45, 56). In this study, SEM framework pathway analyses revealed that older and women participants were associated with a lower number of teeth and poorer nutritional status. However, a machine learning prediction analysis yielded different results (57). The analysis suggested that men have a higher risk of tooth loss compared to women, which was associated with the observation that most smokers within the study cohort were men. We also observed interesting differences in the number of teeth and nutritional status among these ethnic groups, with the Uyghurs group having the fewest number of teeth and the Yi group demonstrating the most compromised nutritional status. Researchers stated that genetic variant might be associated with dental morphology and dental related disease in Uyghurs. The EDARV370A genetic variant was associated with dental morphology among Uyghurs (58), and PAX9 and MSX1 gene mutations were related to non-syndromic oligodontia in Uyghurs (59). Genetics may contribute to the reduced number of residual teeth observed in Uyghurs. Limited evidence showed that cultural beliefs and customs could influence oral health through oral hygiene practices and diet (60). Some ethnic minorities may exhibit inadequate oral hygiene practices, such as utilizing leaves or twigs for dental cleaning instead of conventional toothbrushes (61). Concurrently, due to limited economic resources and educational levels, they may perceive tooth loss as a normal aspect of aging, thereby overlooking the implications of tooth loss on dietary choices and overall health (62). Furthermore, as older individuals experience a reduction in the number of teeth, their chewing ability may diminish, which could adversely affect dietary selection and lead to negative outcomes such as insufficient nutrient intake (63). Yi groups have diverse dietary habits and patterns, with differences between urban and rural areas (64) that would impact nutritional status. However, most research on the poor nutritional status of Yi people has focused on children (65, 66), and more relevant studies need to focus on multi-ethnic older adults in the future.

China is a vast country with numerous ethnic groups. Due to economic, geographical, linguistic, and other factors (67), minority area health human resources are relatively scarce, leading to limited access to healthcare resources (68). Improving the oral health and nutritional status of older adults in minority areas is essential. This study preliminarily explored nutritional status mediated the relationship between the number of teeth and depressive symptoms among older adults residing in western China communities. Future studies should consider long-term longitudinal follow-up to systematically analyze the multidimensional influencing factors on the mental health of multi-ethnic older adults. Nurse-led interprofessional care, addressing both health and social needs could improves depression outcomes in older adult adults (69). Therefore, community caregivers should collaborate with interprofessional teams (such as dental specialists, dietitians, and social workers), focusing on the oral health and nutritional status of older adults and providing older adult-centered mental health care services. Communities could develop personalized oral health care plans for older adults by integrating various oral health care practices. This may include regular home visits from professional dentists to conduct oral examinations and provide guidance on proper toothbrushing techniques. Moreover, it is essential to focus on the nutritional status of the older population. The integration of nutritionists into community healthcare teams could enhance the formulation of diverse and age-appropriate dietary strategies. These potential intervention strategies are necessary for the prevention of depression.

### 4.1 Limitations

This study has several limitations. First, the cross-sectional design precludes the determination of causality in the correlation between the number of teeth and depressive symptoms mediated by nutritional status. Second, lack of detailed oral health variables such as periodontitis, chewing ability, tongue pressure, and swallowing. Third, GDS-15 functions as a screening tool for depression rather than a diagnosis tool. Potential bias existed in the analysis, so cautiously interpreting the results was necessary. Longitudinal data is essential to confirm the link between the number of teeth, nutritional status, and depressive symptoms. Future research should employ objective measures for assessing nutritional status, perform thorough examinations of oral health, and utilize clinically structured assessments for diagnosing depression.

## 5 Conclusion

In summary, our study revealed significant association between the number of teeth, nutritional status, and depressive symptoms in multi-ethnic older adults in western China. It was found that nutritional status plays a mediating role in the relationship between the number of teeth and depressive symptoms. Therefore, it is important for community caregivers to prioritize the overall oral health and nutritional status of older individuals to mitigate the risk of depression.

## Data Availability

The raw data supporting the conclusions of this article will be made available by the authors, without undue reservation.
