# Supplementary material for: Mediation of nutritional status between the number of teeth and depressive symptoms in multi-ethnic older adults from Western China
Source: Front Public Health. 2025 Apr 29;13:1506640. doi: 10.3389/fpubh.2025.1506640 (PMC12069046; doi:10.3389/fpubh.2025.1506640)
Supplement: Supplementary file 1 [file Table_1.docx]

Supplementary Material

# Supplementary Table 1. MNA-SF score as mediator of the effect of teeth number on depressive symptoms.

|  | | Model 1 | | | Model 2 | | |
| --- | --- | --- | --- | --- | --- | --- | --- |
|  |  | *β* | *p* | 95%CI | *β* | *p* | 95%CI |
| MNA-SF score | ACME | -0.0935 | ＜0.001 | -0.1113 to -0.0768 | -0.0592 | ＜0.001 | -0.0762 to -0.0438 |
|  | ADE | -0.0577 | 0.048 | -0.112 to -0.0005 | -0.0883 | 0.006 | -0.1545 to -0.0174 |
|  | Total Effect | -0.1513 | ＜0.001 | -0.2061 to -0.0948 | -0.1475 | ＜0.001 | -0.2145 to -0.0775 |
|  | Prop. Mediated | 0.6189 | ＜0.001 | 0.4447 to 0.9953 | 0.4012 | ＜0.001 | 0.2523 to 0.7775 |

Cl: confidence interval; MNA-SF: Mini Nutrition Assessment-Short Form; ACME, average causal mediation effects (indirect effect); ADE, average direct effects; Prop. Mediated, the mediator variable explains the percentage of the association between the number of teeth and depressive symptoms. Mediation analysis statistic details: In model 1, no covariates were adjusted. In model 2, the covariates included age, gender, ethnic group, educational level, occupation, marital status, longevity of family, denture usage status, type of drinking water, and history of smoking, alcohol use, and drinking tea were adjusted.

# Supplementary Table 2. The summary of structural equation model.

| Pathway | Estimate | SE | z | *P* |
| --- | --- | --- | --- | --- |
| Number of teeth <- Age | -0.4384 | 0.0109 | -40.0761 | ＜0.001 |
| Number of teeth <- Gender | -0.0921 | 0.0109 | -8.4497 | ＜0.001 |
| Number of teeth <- Uyghur | -0.2327 | 0.0115 | -20.2668 | ＜0.001 |
| Number of teeth <- Yi | -0.1105 | 0.0115 | -9.6000 | ＜0.001 |
| Number of teeth <- Zang | -0.1000 | 0.0121 | -8.2749 | ＜0.001 |
| Number of teeth <- Others | -0.0584 | 0.0114 | -5.1103 | ＜0.001 |
| Number of teeth <- Qiang | 0.0007 | 0.0120 | 0.0544 | 0.957 |
| MNA-SF score <- Age | -0.0923 | 0.0134 | -6.8734 | ＜0.001 |
| MNA-SF score <- Gender | -0.0601 | 0.0121 | -4.9771 | ＜0.001 |
| MNA-SF score <- Uyghur | -0.0419 | 0.0130 | -3.2173 | 0.001 |
| MNA-SF score <- Yi | -0.1444 | 0.0128 | -11.3124 | ＜0.001 |
| MNA-SF score <- Zang | -0.0381 | 0.0134 | -2.8505 | 0.004 |
| MNA-SF score <- Others | -0.1062 | 0.0126 | -8.4200 | ＜0.001 |
| MNA-SF score <- Qiang | -0.0257 | 0.0132 | -1.9482 | 0.051 |
| MNA-SF score <- Number of teeth | 0.1341 | 0.0135 | 9.9126 | ＜0.001 |
| Depressive symptoms <- Number of teeth | -0.0259 | 0.0122 | -2.1240 | 0.034 |
| Depressive symptoms <- MNA-SF score | -0.2200 | 0.0122 | -18.0629 | ＜0.001 |

MNA-SF: Mini Nutrition Assessment-Short Form.


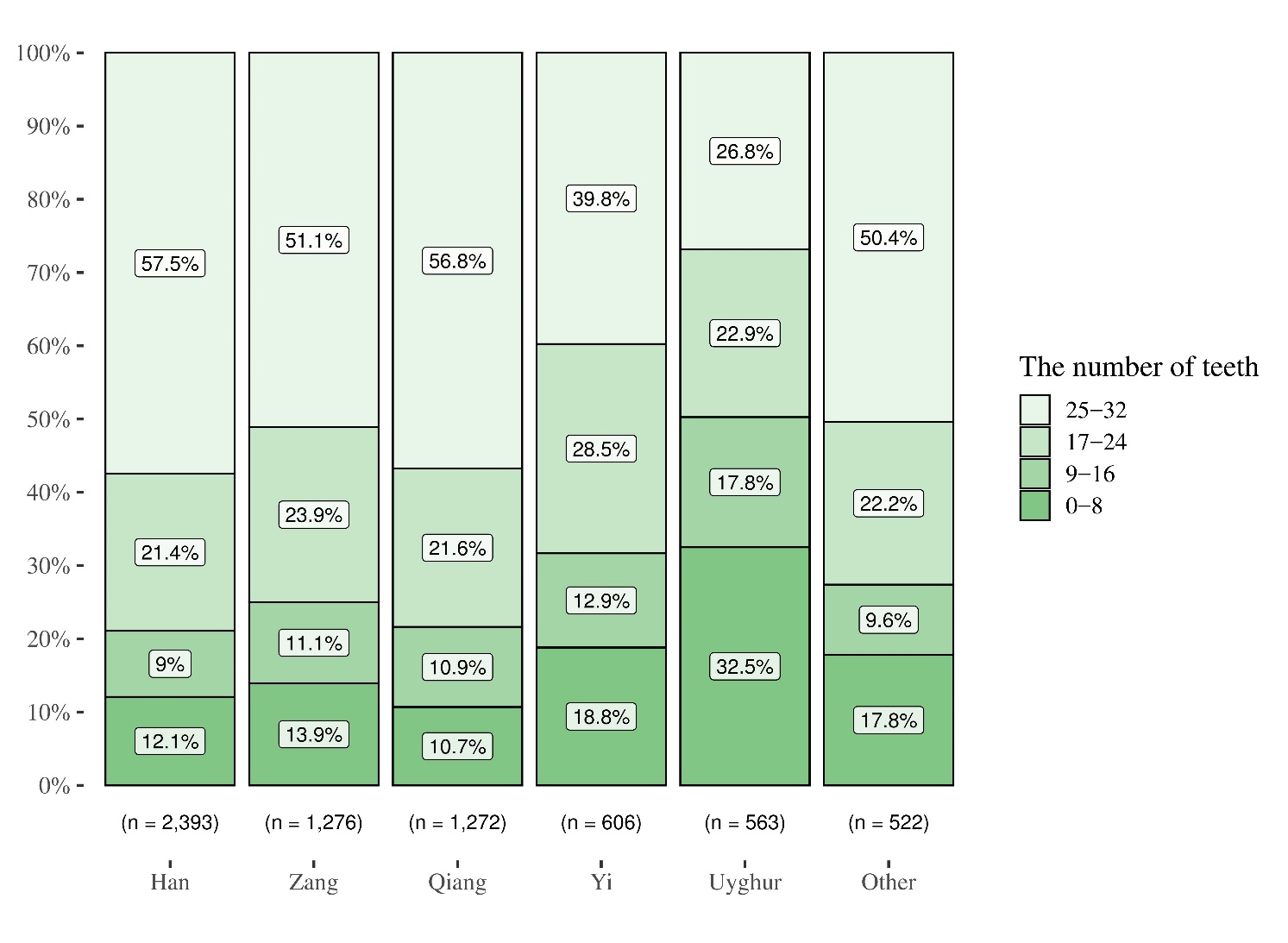


**Supplementary Figure 1.** The distribution of the number of teeth in different ethnic groups. Other ethnic group including the Zhuang, Man, Hui, Mongolian, Tujia, Bai, Khalkhas, Dong, Miao, and Lisu ethnic groups.


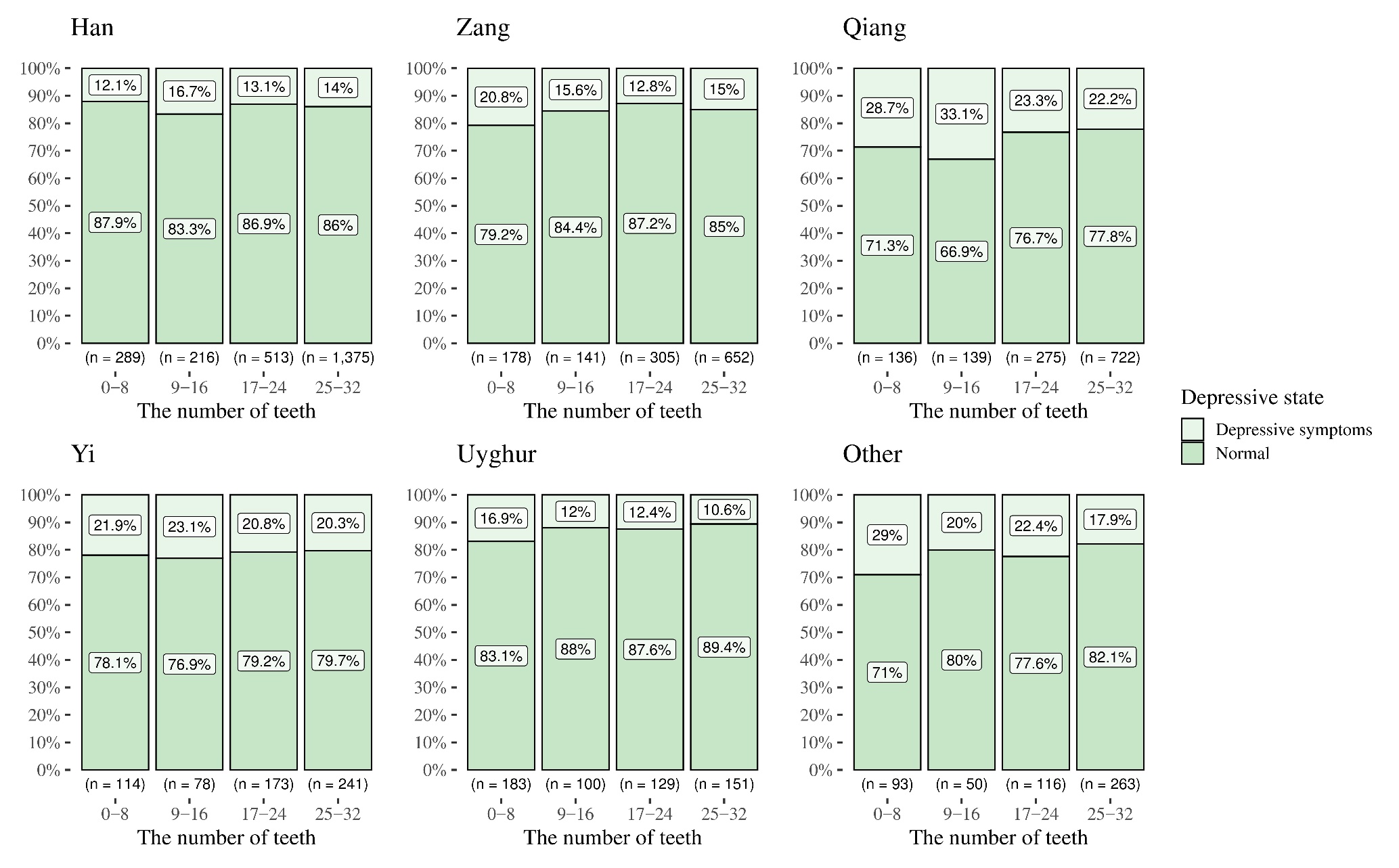


**Supplementary Figure 2.** The proportion of the number of teeth and depressive symptoms in different ethnic groups. Other ethnic group including the Zhuang, Man, Hui, Mongolian, Tujia, Bai, Khalkhas, Dong, Miao, and Lisu ethnic groups.
